# Propensity Score–Matched Analysis of Arthroscopically Assisted Ankle Facture Treatment Versus Conventional Treatment

**DOI:** 10.1177/1071100720969609

**Published:** 2020-12-17

**Authors:** Sebastian F. Baumbach, Marcel Urresti-Gundlach, Mareen Braunstein, Lars Borgmann, Wolfgang Böcker, J. Turner Vosseller, Hans Polzer

**Affiliations:** 1 Department of General, Trauma and Reconstructive Surgery, University Hospital, LMU Munich, Germany; 2 Center for Higher Education, TU Dortmund University, Dortmund, Germany; 3 Department of Orthopaedic Surgery, Columbia University Medical Center, New York, NY, USA

**Keywords:** ankle fracture, arthroscopically assisted fracture treatment, arthroscopy, AORIF, ankle, chondral lesion, microfracture

## Abstract

**Background:**

The aim of this study was to assess the prospective, longitudinal outcome after arthroscopically assisted open reduction and internal fixation (AORIF) and to compare the results with open reduction and internal fixation (ORIF) in complex ankle fractures.

**Methods:**

Acute, closed, bimalleolar equivalent, bimalleolar, or trimalleolar ankle fractures were included. The AORIF cohort was enrolled prospectively. The ORIF group was identified from a retrospective database. The same inclusion and exclusion criteria were applied. The only difference was the additional arthroscopy in the AORIF cohort. The patient-reported outcome measurement (PROM) following AORIF was assessed at 1 and 4 years of follow-up using the Olerud and Molander Ankle Score (OMAS) and Tegner activity scale (TAS). The AORIF cohort was propensity score matched (nearest-neighbor matching) to the ORIF database. The OMAS and Foot and Ankle Ability Measure (FAAM) were compared between the resulting groups. Nonparametric statistics were applied; values are presented as median (interquartile range). Twenty-six AORIF patients had a prospective 4-year follow-up.

**Results:**

No significant differences (1 year vs 4 years) were identified for the OMAS (90 [10] vs 90 [11]) and TAS (4 [2] vs 5 [2]). The severity of the cartilage lesions (International Cartilage Repair Society [ICRS] grade <4 vs ICRS of 4) had no significant influence on the PROMs. Twenty-five patients per cohort (AORIF vs ORIF) were matched. The OMAS (90 [13] vs 75 [40]; *P* = .008) and FAAM Activities of Daily Living (ADL; 96 [11] vs 88 [30]; *P* = .034) revealed significantly better outcomes for AORIF. More patients in the AORIF cohort returned to sport (96% vs 77%; *P* = .035), with a higher FAAM Sports score (88 [37] vs 56 [47]; *P* = .008).

**Conclusion:**

AORIF for complex ankle fractures led to consistently good to excellent results. The propensity score–matched analysis revealed a significantly better outcome 4 years after surgery for AORIF compared with ORIF.

**Level of Evidence:**

Level III, retrospective comparative study.

## Introduction

Ankle fractures are among the most common injuries to the lower extremity. Many of these fractures are treated by open reduction and internal fixation (ORIF) in order to achieve an anatomic reduction and potentially decrease the risk of posttraumatic arthritis. Although the quality of reduction correlates with the postoperative result,^27^ it does not necessarily guarantee a good outcome. In a current review of 1822 patients, only 79% of the patients with an anatomically reduced ankle fracture achieved a good to excellent outcome after a mean follow-up of 5 years.^28^ In another study, only 52% had a good or very good result 10 years following ORIF of an ankle fracture.^10^

A growing number of authors attribute these inferior results to the presence of traumatic chondral lesions.^3,12,14,18,27^ Assisted open reduction and internal fixation (AORIF) allows for the identification and treatment of these lesions. Immediate treatment with microfracture appears to be superior when compared with debridement only.^11^ Furthermore, additional arthroscopy apparently does not appear to increase the complication rate associated with ankle fracture surgery.^34^ Despite these considerations, AORIF is far from being the standard of care. In a current analysis of 32307 patients surgically treated for ankle fractures, only 1% received additional arthroscopy.^34^

The number of studies comparing AORIF with ORIF is limited.^13^ To the authors’ knowledge, only 4 comparative studies have been published, all including patients with simple isolated fractures of either the fibula^12,29,31^ or the medial malleolus^33^ only. Still, it has been shown that the complexity of an ankle fracture has a significant influence on the outcome^15,17,26,28^ and that the number and severity of chondral injuries increase with the complexity of the fracture.^1,5,14,16,18,27^ Therefore, the focus on more simple fractures in these studies limits the assessment of the impact that arthroscopy could have. It is possible that the positive impact of AORIF in the treatment of ankle fractures would increase with the complexity of the fracture.

The aim of this study was to assess the prospective, longitudinal outcome after AORIF and to compare the results to regular ORIF in complex ankle fractures.

## Methods

### Study Design

The study was approved by the local ethics committee and was split into 2 parts:

1. Prospective, longitudinal analysis of patient-reported outcome measurements (PROMs) 1 and 4 years following AORIF of complex ankle fractures
2. Propensity score–matched analysis comparing the AORIF cohort to patients who were conventionally treated (ORIF) based on our ankle fracture database

### Patient Selection

**Figure 1.**
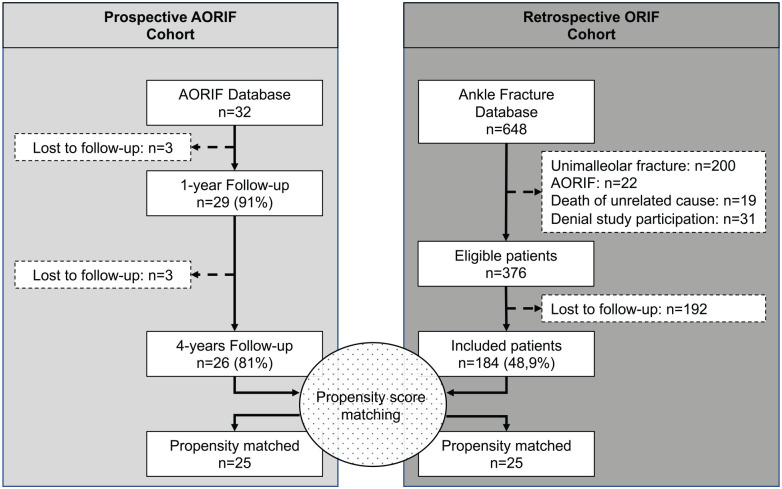
Patient selection flowchart. AORIF, assisted open reduction and internal fixation; n, number of patients; ORIF, open reduction and internal fixation.

The study had 2 patient cohorts. The patient selection process is depicted in Figure 1. For the prospective AORIF cohort, the detailed selection process, intraoperative findings, and PROMs for the AORIF cohort after 1 year have been published previously.^5^ In brief, all patients presenting to our department between May 2013 and December 2014 older than 17 years of age, with an acute bimalleolar equivalent, bimalleolar, or trimalleolar ankle fracture, which we considered complex fractures, were treated by AORIF. The inclusion and exclusion criteria are depicted in Table 1.

**Table 1.** Inclusion and Exclusion Criteria for Both the Prospectively Enrolled Patients for AORIF and the Retrospectively Selected Comparison Cohort (ORIF).

Inclusion	Exclusion
Unimalleolar fracture + syndesmotic disruption = bimalleolar equivalent	Isolated unimalleolar fracture
Bi- or trimalleolar fracture ± ligamentous injury	Pilon fracture
>17 years	Open fracture
Date of injury ≤14 days to surgery	Mental illness, noncompliance, pregnancy
Written informed consent	Multiple injuries

Abbreviations: AORIF, assisted open reduction and internal fixation; ORIF, open reduction and internal fixation.

For the second part of the study, propensity score matching was conducted between the previously outlined AORIF cohort and a retrospective cohort treated by ORIF only (ORIF cohort), identified from the department’s ankle fracture database. This ankle fracture database has been described in detail previously.^2^ Any patient from the department’s ankle fracture database who was treated by ORIF and met the same inclusion and exclusion criteria applied for the AORIF cohort was eligible for the matching process (Table 1). These patients provided the basis for the propensity score matching.

### Propensity Score Matching

Propensity score matching is a statistical method to limit the treatment selection bias on the estimation of causal treatment effects in retrospective cohort studies.^8^ It aims to overcome the covariate imbalance prevalent in observational studies so that causal estimates of treatment outcome relationships can be made. The 7 covariates included in the model were age, sex, American Society of Anesthesiologists (ASA) class, body mass index (BMI), side, number of malleoli fractured, and follow-up. Based on these parameters, the authors aimed to generate pairs of treated (AORIF) and untreated (ORIF) subjects using logistic regression-based propensity scores applying nearest-neighbor matching. A predefined caliper width of 0.1 without case replacement was used. This approach resulted in 25 pairs of treated (AORIF) and untreated (ORIF) subjects. Propensity score matching was calculated using Python Software Foundation, Python Language Reference, version 3.7 (https://www.python.org).

### Operative Technique

#### AORIF Cohort

The treatment strategy for the patients included in the prospective AORIF cohort has been outlined in detail previously.^4,5^ In summary, diagnostic ankle arthroscopy was performed first. All intra-articular pathologies were documented, and chondral lesions were graded as recommended by the International Cartilage Repair Society (ICRS).^6^ Loose bodies were removed, grade 2 and 3 chondral lesions (according to ICRS) were debrided, and a chondroplasty was performed. Grade 4 lesions were treated by chondroplasty and microfracture. ORIF of the ankle fracture was then performed as previously detailed.^5^ After osteosynthesis, the arthroscope was reinserted to assess the adequacy of the reduction.

#### ORIF Cohort

ORIF for the authors’ database has been described in detail previously.^2^ In brief, the lateral and medial malleolar fractures were treated by ORIF using lag screws and/or plate osteosynthesis in accordance with AO principles.^24^ Fractures of the posterior malleolus were treated per the surgeon’s preference, either not fixed (nondisplaced fractures), fixed by closed reduction and fixation using percutaneous screw(s) from anterior to posterior, or fixed by ORIF under direct visualization. In the case of syndesmotic instability, the distal tibiofibular joint was reduced and fixed either by a suture-button or transsyndesmotic screw.

### Aftercare

The postoperative protocol was identical for all patients in both cohorts. The ankles were not immobilized, and patients were advised to conduct 20-kg partial weightbearing for 6 weeks. Active range of motion exercises were encouraged immediately postoperatively. After 6 weeks, follow-up radiographs were conducted, and the patients were allowed to proceed to full weightbearing over a 2-week period.

### Data Assessed

Standard demographics (age and sex), BMI, ASA class, and complications were retrieved from the patients’ charts. All fractures were classified according to the AO classification system. The quality of reduction was assessed for each fracture separately (<2 mm = 1 point; ≥2 mm = 2 points for dislocation/gap in any plane),^30^ and the point average was calculated for each patient. Whenever possible, the degree of reduction was assessed on postoperative CT scans. All data for the AORIF cohort were collected prospectively, while the ORIF cohort was assessed retrospectively. The same variables were collected in the same way for both cohorts. The PROMs for both groups were assessed either during a follow-up visit, during a telephone interview, or by a questionnaire sent by mail. For both cohorts, the PROMs assessed after 4 years were the Olerud and Molander Ankle Score (OMAS)^22^ and the Foot and Ankle Ability Measure (FAAM).^19^ The Tegner activity scale (TAS)^31^ was assessed for the AORIF cohort only. The FAAM score is divided into 2 subscales, the 21-item Activities of Daily Living (ADL) and the 8-item Sports subscale. For the ORIF cohort, the OMAS and FAAM were retrieved from the authors’ ankle fracture database.

### Outcome Parameters

The primary outcome parameters were the OMAS and FAAM. Secondary outcome parameters for the longitudinal analysis of the AORIF cohort included the TAS, and the outcomes per the severity of cartilage lesions (ICRS grade <4 versus ICRS grade 4).

### Statistics

The Shapiro-Wilk test revealed that neither part of the study had normally distributed data. Data are presented as median (interquartile range).

Nonparametric testing was applied, including the related-samples Wilcoxon signed-rank test, related-samples Friedman’s 2-way analysis of variance by ranks, independent-samples Mann-Whitney *U* test, independent-samples Kruskal-Wallis test, and Fisher’s exact test. Due to multiple testing, a Bonferroni alpha-level correction was performed for the secondary outcome parameters, setting the level of significance at *P* < .005.

## Results

### AORIF at 1 Year Versus 4 Years

The demographics of the AORIF 1-year follow-up study have been published previously.^5^ In brief, out of 89 patients, 32 were eligible and a 1-year PROM follow-up was available for 29 patients. Of that group, 26 patients (81%) were available for the 4-year follow-up. General demographics, ASA class, fracture characteristics, syndesmosis instability, quality of reduction, and complications are summarized in Table 2. Eighty-nine percent of patients had a cartilage lesion, with a median ICRS grade of 2, and 35% suffered a full-thickness lesion (ICRS grade 4). Final arthroscopic inspection, following osteosynthesis, demonstrated anatomical reduction in all cases.

**Table 2.** General Demographics, ASA Class, Fracture Characteristics, Syndesmosis Instability, and Complications for the Propensity-Matched 4-Year Follow-Up AORIF and ORIF Cohorts.

	AORIF		ORIF		*P* value
Total no. of patients	25		25		
Median age, y (IQR)	46 (28)		53 (22)		.154
Sex, % female	60		56		>.99
Side, % left	40		40		>.99
Median BMI (IQR)	25 (8)		28 (6)		.232
Median ASA class (IQR)	2 (1)		2 (1)		.467
Fracture type					.714
Bimalleolar equivalent	B1.2: *n* = 1 B1.3: *n* = 2 C3.2: *n* = 1	16% (*n* = 4)	A2.1: *n* = 1 B2.1: *n* = 1 C1.1: *n* = 1	12% (*n* = 3)	
Bimalleolar	A3.3: *n* = 1 B2.3: *n* = 3 B3.1: *n* = 2	20% (*n* = 6)	B2.2: *n* = 2 B2.3: *n* = 3 B3.1: *n* = 3 Weber C + PM: *n* = 2	32% (*n* = 10)	
Trimalleolar	B3.2: *n* = 1 B3.3: *n* = 12 C2.3: *n* = 2	64% (*n* = 15)	B3.2: *n* = 2 B3.3: *n* = 7 C1.3: *n* = 1 C2.3: *n* = 2	56% (*n* = 12)	
Quality of reduction^a^	1.0 ± 0.1		1.1 ± 0.3		.369
Major complications, %	12		8		.794
Median follow-up (IQR)	4.4 (0.8)		4.0 (3.4)		.064

Abbreviations: AORIF, assisted open reduction and internal fixation; ASA, American Society of Anesthesiologists; BMI, body mass index; IQR, interquartile range: ORIF, open reduction and internal fixation.

a The quality of reduction was assessed for each fracture separately (<2 mm = 1 point; ≥2 mm = 2 points for dislocation/gap in any plane),^30^ and the point average was calculated for each patient. Due to the high degree of quality of reduction, the values are given as mean ± standard deviation.

**Figure 2.**
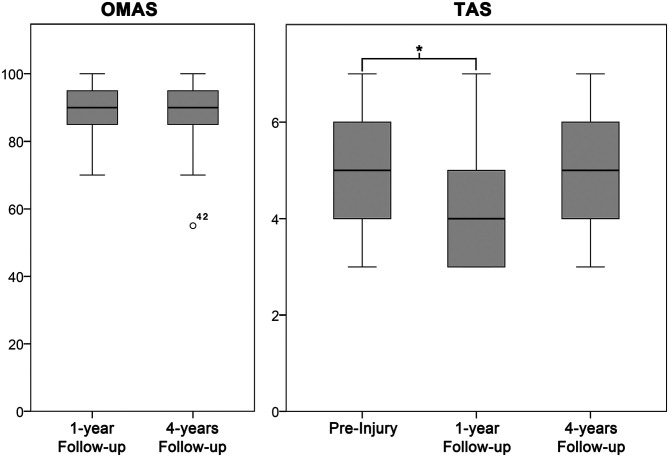
Boxplots of the longitudinal Olerud and Molander Ankle Scores (OMASs) and Tegner activity scale (TAS) scores. All boxplots were generated with SPSS. The median is shown by the bold lines, and the first and third quartiles by the gray boxes. The whiskers represent 1.5 times the interquartile range, and circles are outliers.

The median follow-up was 4.4 (0.9) years. No significant differences could be detected for the OMAS when comparing the 1-year (90 [10]) and 4-year (90 [11]) follow-ups (*P* = .962) (Figure 2). A significant difference (*P* = .001) was observed for the longitudinal follow-up of the TAS between preinjury (5 [2]) and the 1-year (4 [2]) and 4-year (5 [2]) follow-ups (Figure 2). The subgroup analysis looking at the influence of the severity of the cartilage lesion (ICRS grade <4 vs ICRS grade 4) revealed no significant differences within each follow-up or between the 1- and 4-year follow-ups (Table 3).

**Table 3.** Subgroup Analysis Comparing the PROMs of the AORIF Cohort per the Degree of Cartilage Lesion.^a^

		ICRS grade <4	ICRS grade 4	*P* value
OMAS	1-year FU	90 (10)	85 (15)	>.99
	4-year FU	90 (11)	90 (15)	.672
	*P* value	0.655	0.931	
TAS	Preinjury	5 (3)	5 (2)	>.99
	1-year FU	4 (3)	4 (2)	.958
	4-year FU	5 (2)	5 (2)	.751
	*P* value	.314	.502	

Abbreviations: AORIF, assisted open reduction and internal fixation; FU, follow-up; ICRS, International Cartilage Repair Society; OMAS, Olerud and Molander Ankle Score; PROM, patient-reported outcome measurement; TAS, Tegner activity scale.

a Data are presented as median (IQR).

### AORIF Versus ORIF

The authors’ ankle fracture database included 648 patients, 376 of which were eligible (Figure 1). A prospective follow-up of the OMAS and FAAM was available for 184 (48.9%) patients. This population of 184 patients formed the basis for the propensity score matching. Twenty-five patients of both cohorts (AORIF and ORIF) were included in the final analysis (Table 2).

**Figure 3.**
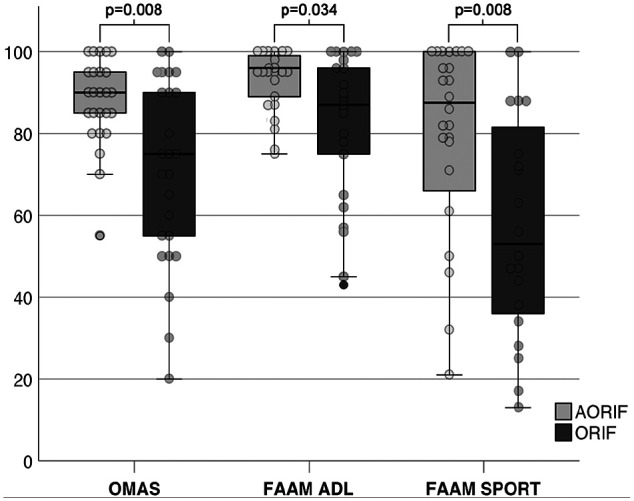
Boxplots and dot plots comparing the patient-reported outcome measurements of the AORIF (assisted open reduction and internal fixation) to the ORIF (open reduction and internal fixation) cohort. All boxplots were generated with SPSS. The median is shown by the bold lines, and the first and third quartiles by the gray boxes. The whiskers represent 1.5 times the IQR, and circles are outliers. ADL, Activities of Daily Living; FAAM, Foot and Ankle Ability Measure; OMAS, Olerud and Molander Ankle Score.

The OMAS and FAAM are illustrated in Figure 3. Based on the propensity score matching, all outcome measures showed significantly better results for the AORIF compared with the ORIF cohort. The differences were 15 points for the OMAS, 8 points for the FAAM ADL, and 32 points for the FAAM Sports. Moreover, more patients returned to sports in the AORIF compared with the ORIF cohort (96% vs 84%).

## Discussion

The aim of this study was first to assess the prospective, longitudinal outcome after AORIF and then to compare the results with ORIF in complex ankle fractures. The prospective 4-year follow-up of the AORIF group revealed good to excellent OMASs and TAS scores. Further, no deterioration of these outcomes was observed between the 1-year and 4-year follow-ups. The severity of the cartilage lesion had no significant effect on outcome at any time point. Twenty-five patients per cohort (AORIF and ORIF) were propensity matched. The AORIF cohort showed significantly better scores for the OMAS and FAAM as well as a significantly higher rate of patients returning to sports.

The vast majority of previous studies reporting on the results following AORIF in ankle fractures included simple fracture types only.^7^ Up to now, only 4 comparative studies, all limited to unimalleolar fractures, have been published.^12,29,31,32^ Longitudinal follow-up data were not available in these studies. This is the first cohort study reporting on prospective, longitudinal data on AORIF in complex fractures with a concomitant comparison of these patients with patients that had ORIF alone.

### AORIF at 1 Year Versus 4 Years

The prospective analysis of the results 1 and 4 years following AORIF showed no difference in the OMAS, a foot and ankle function score specifically designed to assess results after ankle fractures. The clinical results (OMAS of 90 points) at both time points were good to excellent. These results are very promising when compared with studies on ORIF only. One study noted a mean OMAS of 75 points following ORIF for isolated fibula fractures in 69 patients after 5 years,^25^ while another^17^ reported a mean OMAS of 71 points after 2 years in 74 patients also with isolated malleolus fractures only. Ponzer et al^23^ found a mean OMAS of 84 points, with only 36% of the patients achieving a complete recovery, while 64% had sports- or work-related problems, after 2 years following ORIF of AO type B fractures. The current study also showed that the TAS, a general physical activity scale, was significantly decreased after 1 year but improved to the preinjury level at the 4.4-year follow-up. These findings are in good agreement with the high rate of patients returning to sports in the AORIF cohort (96%). Consequently, AORIF achieved excellent clinical outcomes, as well as allowing for a high rate of return to sport. Interestingly, the rehabilitation period appears to exceed 1 year.

The additional arthroscopy allows the surgeon to diagnose and treat intra-articular injuries, especially cartilage lesions. These are, at least in part, thought to be responsible for unfavorable outcomes following ORIF. The subgroup analysis in the current study noted that there was no significant difference in outcome between full-thickness cartilage lesions (ICRS grade 4) and less severe lesions (ICRS < grade 4), either within each follow-up or between the 1- and 4-year follow-ups (Table 3). This is particularly promising, as previous studies have suggested that deep chondral lesions are an independent predictor of an inferior outcome.^9,27^ It therefore can by hypothesized that sufficient treatment of severe chondral lesions (ICRS grade 4; microfracture) might be able to decrease the likelihood of symptoms resulting from these lesions in the medium term (ie, 4 years). This finding is in line with previous studies showing better results for microfracture of full-thickness lesions compared with debridement only.^11^

### AORIF Versus ORIF

The propensity score matching analysis noted that AORIF resulted in significantly better outcome with both the OMAS and FAAM. Moreover, the difference in scores between the 2 cohorts (OMAS: 15 points; FAAM: 8 points) exceeded the minimal detectable change of both scores (OMAS: 10 points;^20,22^ FAAM: 4-7 points^21^) The distribution of the results seems to suggest more consistent results in favor of AORIF. Figure 3 compares the outcome variables per the 2 treatment groups. Interestingly, the interquartile ranges were considerably narrower in the AORIF compared with the ORIF cohort (OMAS: 11 vs 25 points; FAAM ADL: 10 vs 26 points). Whereas the smaller interquartile ranges in the AORIF cohort does argue for the reproducibility of the results, the wide interquartile range in the ORIF cohort might be explained by the untreated intra-articular pathologies. These encouraging results were further highlighted by a significantly higher return to sport rate in the AORIF cohort (96% vs 77%; *P* = .035). Yet, it remains unknown whether the nonarthroscopic group had comparable cartilaginous lesions to the AORIF group. Nevertheless, this is a problem of any study comparing ORIF with AORIF. Next to intra-articular pathologies, numerous other parameters are known to affect the outcome of surgically treated ankle fractures. Some of the most pronounced factors are age, sex, ASA class, BMI, side, number of malleoli fractured, and follow-up. Using propensity score matching, a logistic regression, nearest-neighbor matching, was applied to control for all of these parameters. The propensity score matching thereby allows us to generate comparable groups and estimate the effect of the intervention, that is, additional arthroscopy.

As outlined above, the 4 comparative studies currently published are all limited to unimalleolar fractures only.^12,29,31,32^ This is not an insignificant limitation, as it is known that the number and severity of chondral lesions increase with the complexity of the fracture.^18,27^ The data presented in these 4 studies are heterogeneous. One study on a rather small sample size found no significant differences for the SF-36 and the MODEMS Lower Extremity Foot and Ankle Score after 21 months.^32^ In contrast, a larger study revealed significantly better results for the AORIF cohort (AOFAS: 91 vs 88).^29^ However, the differences observed, although statistically significant, did not meet the minimal detectable change, and the legacy outcome metric used is not validated. For the 2 retrospective cohort studies, one found no significant differences in the PROMIS (Patient-Reported Outcomes Measurement Information System), visual analog scale score, and OMAS between the 2 cohorts,^12^ whereas the other did find significantly superior results for the OMAS for AORIF when compared with ORIF (OMAS: 92 vs 86 points).^33^

### Limitations

There are several limitations to this study. The most pronounced one is that the ORIF cohort, in contrast to the AORIF cohort, was assessed retrospectively. In order to reduce the selection bias due to differences in the covariates, a propensity score method was applied. The propensity scores balance the baseline distribution of covariates between the AORIF and ORIF groups and thereby reduce a possible matching bias.^8^ Still, one has to be aware that it does not generate identical groups. This explains why the follow-up in the ORIF cohort was shorter and showed a greater interquartile range. Moreover, although the model included the number of malleoli fractured, the 2 groups compared varied not only per the number of malleoli fractured but also per the AO classification. Still, both the shorter follow-up period (4.4 vs 4.0 years) and the more moderate fracture severity (bimalleolar: 20% vs 32%; trimalleolar: 64% vs 56%) are advantages for the ORIF cohort. Next, we could not control for further factors associated with fracture severity, such as the number of fragments per fracture or intra-articular lesions in the ORIF cohort. Therefore, larger-scaled, single-blinded randomized controlled trials on complex ankle fractures with sufficient follow-up are needed. Further, although not reported previously, both outcome scores, the OMAS and FAAM ADL, showed ceiling effects, resulting in a nonnormal distribution. Therefore, further differentiation of patients with good and very good outcomes might have been hindered. Finally, a priori power analysis was not conducted. A post hoc power calculation (2-tailed; alpha, 0.05) resulted in a power of 86.2% for the OMAS and a power of 98.5% for the FAAM ADL.

Despite these limitations, this is the first comparative study including complex ankle fractures. Furthermore, the follow-up period was much longer compared with previous AORIF studies. A further strength is the application of validated PROMs for both the longitudinal and the comparative study part.

### Conclusion

AORIF for complex ankle fractures led to consistently good to excellent results after 1 and 4 years. This was independent of the severity of the cartilage lesion, with full-thickness lesions being treated by microfracture. Moreover, the propensity score–matched analysis revealed significantly better outcomes 4 years after surgery for AORIF compared with ORIF.

## Supplemental Material

sj-pdf-1-fai-10.1177_1071100720969609 – Supplemental material for Propensity Score–Matched Analysis of Arthroscopically Assisted Ankle Facture Treatment Versus Conventional Treatment Click here for additional data file. Supplemental material, sj-pdf-1-fai-10.1177_1071100720969609 for Propensity Score–Matched Analysis of Arthroscopically Assisted Ankle Facture Treatment Versus Conventional Treatment by Sebastian F. Baumbach, Marcel Urresti-Gundlach, Mareen Braunstein, Lars Borgmann, Wolfgang Böcker, J. Turner Vosseller and Hans Polzer in Foot & Ankle International
